# Does oral sodium bicarbonate therapy improve function and quality of life in older patients with chronic kidney disease and low-grade acidosis (the BiCARB trial)? Study protocol for a randomized controlled trial

**DOI:** 10.1186/s13063-015-0843-6

**Published:** 2015-08-01

**Authors:** Miles D. Witham, Margaret M. Band, Roberta C. Littleford, Alison Avenell, Roy L. Soiza, Marion E. T. McMurdo, Deepa Sumukadas, Simon A. Ogston, Edmund J. Lamb, Geeta Hampson, Paul McNamee, for the BiCARB study group

**Affiliations:** Medical Research Institute, University of Dundee, Ninewells Hospital, Dundee, DD1 9SY UK; Tayside Clinical Trials Unit, University of Dundee / NHS Tayside, Dundee, UK; Health Services Research Unit, University of Aberdeen, Aberdeen, UK; Epidemiology and Biostatistics Unit, University of Dundee, Dundee, UK; Department of Clinical Biochemistry, East Kent NHS Trust, Canterbury, UK; Department of Chemical Pathology, Guy’s and St Thomas’ Hospitals, London, UK; Health Economics Research Unit, University of Aberdeen, Aberdeen, UK

**Keywords:** Older, Bicarbonate, Metabolic acidosis, Physical function, Quality of life, Chronic kidney disease, Randomised controlled trial

## Abstract

**Background:**

Metabolic acidosis is more common with advancing chronic kidney disease, and has been associated with impaired physical function, impaired bone health, accelerated decline in kidney function and increased vascular risk. Although oral sodium bicarbonate is widely used to correct metabolic acidosis, there exist potential risks of therapy including worsening hypertension and fluid overload. Little trial evidence exists to decide whether oral bicarbonate therapy is of net benefit in advanced chronic kidney disease, particularly in older people who are most commonly affected, and in whom physical function, quality of life and vascular health are at least as important outcomes as decline in renal function.

**Methods/Design:**

BiCARB is a multi-centre, double-blind, placebo controlled, randomised trial evaluating the clinical and cost-effectiveness of oral sodium bicarbonate in the management of older people with chronic kidney disease and severely reduced glomerular filtration rate (GFR) who have a mild degree of metabolic acidosis. The trial will recruit 380 patients from renal, Medicine for the Elderly, and primary care services across centres in the United Kingdom. Male and female patients aged 60 years and older with an estimated glomerular filtration rate of <30 mL/min/1.73 m^2^, not on dialysis, and with serum bicarbonate concentrations <22 mmol/L will be eligible for participation. The primary clinical outcome for the trial is the between-group difference in the Short Physical Performance Battery score at 12 months. Secondary outcomes include muscle strength, quality of life measured using the EQ-5D score and KDQoL tools, cost effectiveness, renal function, presence of albuminuria and blood pressure. Markers of bone turnover (25-hydroxyvitamin D, 1,25-hydroxyvitamin D, tartrate-resistant acid phosphatase-5b and bone-specific alkaline phosphatase) and vascular health (B-type natriuretic peptide) will be measured. Participants will receive a total of 24 months of either bicarbonate or placebo. The results will provide the first robust test of the overall clinical and cost-effectiveness of this commonly used therapy in older patients with severely reduced kidney function.

**Trial registration:**

www.isrctn.com; ISRCTN09486651, registered 17 February 2012

## Background

Chronic Kidney Disease (CKD) is common: 5 % of the population of the United Kingdom has an estimated glomerular filtration rate (eGFR) of <60 mL/min/1.73 m^2^ (that is, GFR stages 3 to 5), and rates in people over 70 are five times higher than this [1]. Population-based estimates suggest a prevalence of advanced CKD (GFR 15 to 29 mL/min/1.73 m^2^) of approximately 2 % in those aged 70 and over [2], and approximately 20 % of such patients will have a degree of metabolic acidosis, with rates increasing as renal function declines [3]. The introduction of routine eGFR reporting by laboratories has increased the number of older patients diagnosed with CKD, and bicarbonate is often used to treat older people with low serum bicarbonate concentrations. Trial evidence to underpin the effectiveness and safety of this intervention is, however, lacking [4], and this lack of evidence is reflected in current guidelines, including the Kidney Disease Improving Global Outcomes (KDIGO), Scottish Intercollegiate Guidelines Network (SIGN) and UK National Institute for Health and Care Excellence (NICE) guidelines, which either give guidance based on expert consensus without underpinning evidence or note that it is not currently possible to make an evidence-based recommendation regarding correction of mild to moderate metabolic acidosis in CKD. There is variability in clinical practice with regard to the use of bicarbonate therapy in patients with CKD and mild acidosis. Measurement and correction of acidosis is often part of standard care for patients managed under renal services but is less common for patients managed by primary care or Medicine for the Elderly services.

**Fig. 1:**
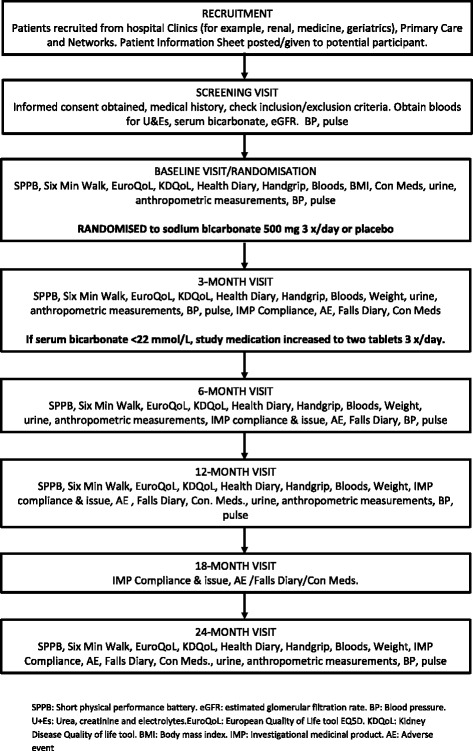
Participant flow through trial

Older people are the group most likely to have CKD, but despite this, few trials have included many older people with CKD. Older patients with CKD invariably have a wide range of comorbid diseases. A narrow focus on kidney disease indices alone is therefore unlikely to reflect what is important to the patient, and the narrow focus on single diseases is a justifiable criticism of many guidelines, which when applied to older people with multi-morbidity may not provide a net gain in health or wellbeing [5, 6]. It is thus important to measure a range of outcomes of relevance to older people, seeking evidence of both benefit and harm.

### Potential benefits of bicarbonate therapy

Chronic acidosis has been associated with the following biological effects [7]: muscle weakness [8], leading to fatigue and falls; weight loss and increased protein breakdown [9]; increased bone resorption leading to osteoporosis [10]; worsening of hyperkalemia leading to arrhythmias; impaired glucose homeostasis [11]; and deranged thyroid function [12], with some evidence that bicarbonate therapy can reverse these physiological derangements. Acidosis may also cause the following, although the existing evidence is less clear: acceleration of decline in renal function [13, 14]; worsening cardiac function, leading to decompensated heart failure; and worsening of cardiovascular disease [7].

### Potential adverse effects of bicarbonate therapy

Bicarbonate is awkward to take, as tablets are large, and multiple tablets usually need to be taken. Sodium bicarbonate contains 6 mmol of sodium per 500 mg, which could lead to increased blood pressure (50 mmol of sodium per day has been projected to increase systolic blood pressure by 3.6 to 5.6 mmHg) [15] and fluid overload. Observational studies suggest that higher serum bicarbonate is associated with an increased risk of heart failure in patients with CKD [16]. There is also a possible effect on vascular calcification [7]; raising the blood pH may make calcium and phosphate less soluble, thus promoting precipitation of calcium phosphate within vessel walls. Abdominal discomfort and bloating are recognized side effects listed in the British National Formulary (BNF), perhaps via generation of carbon dioxide in the gut.

### The need for patient-centred outcomes in older people with chronic kidney disease

An approach examining function and quality of life is therefore essential to test whether bicarbonate intervention in older people has overall worthwhile benefit. Physical function and quality of life are the outcomes that older people themselves consider to be the most important [17] as evidenced by a Delphi consensus exercise involving both older patients and clinicians caring for older people, with postponement of death being a less important goal of therapy.

Our approach in designing this study was therefore to focus first and foremost on indices of physical and psychosocial function and quality of life, underpinned by measures of muscle function that correlate with daily activities and which predict future dependence. Added to these are a series of outcomes examining the effect of the intervention on the major associated disease states - renal function, bone health and cardiovascular health, as well as outcomes designed to quantify the side effects and risks including hypertension and heart failure. A comprehensive health economic analysis is integrated into the trial to provide evidence of cost-effectiveness to underpin policy and guideline production.

### Existing trial evidence

In the preparation of this trial, literature searches of MEDLINE, EMBASE and the Cochrane Library were performed, using the search terms: older, elderly, acidosis, kidney disease, CKD, NaHCO3, and bicarbonate. Searches were augmented by checking the reference lists of articles known to be highly relevant and relevant review articles. No systematic reviews were found addressing this topic at the time of grant preparation; since commencing the trial, a systematic review [18] has been published which suggests that bicarbonate therapy may improve eGFR (by 3.2 mL/min/1.73 m^2^), reduce progression to end-stage renal failure, but increase diastolic blood pressure (by 2.8 mmHg). However, only three trials of long-term (>2 months) therapy were included, with only 250 patients enrolled, and one trial [19] enrolled patients with a mean eGFR of 75 mL/min/1.73 m^2^ and mean baseline serum bicarbonate of 26 mmol/L.

A preliminary, single-centre randomized controlled trial comparing bicarbonate supplementation with placebo showed a promising reduction in the rate of deterioration of renal function with bicarbonate supplementation [13], as well as improvements in muscle mass measured by mid-arm muscle circumference. However, participants in this trial were younger (mean age 55 years), and the trial did not attempt to measure patient-centred outcomes such as physical function and quality of life, and did not evaluate the impact of bicarbonate therapy on markers of bone and vascular health.

### The imperative for the BiCARB trial

Chronic kidney disease is common and affects many older people. The accompanying acidosis may worsen the muscle weakness that affects many older people, which is a key risk factor for death and institutionalization [20, 21]. Both falls and osteoporosis lead to 60,000 hip fractures per year in the UK, with 30 % mortality at 1 year [22] and frequent institutionalization. The cost of falls alone has been estimated to exceed £1 billion per year in the UK [23]. Although not all older people with CKD progress to end-stage renal failure requiring dialysis, the effect on quality of life and cost burden from dialysis is considerable: between £15000 and £35000 per patient per year, depending on modality and setting of dialysis [24]. Finally, cardiovascular disease is the leading cause of hospitalization and death in older people and is responsible itself for one-half to one-third of the decline in physical function seen with age [25]. Thus, an intervention that successfully reverses acidosis in this older population may be able to simultaneously improve multiple important, associated comorbidities in older people, with consequent improvements in function and quality of life, as well as potential reductions in hospitalization and later institutionalization. It is against this background that the UK National Institute for Health Research (NIHR) Health Technology Assessment programme commissioned this trial to examine the effectiveness of oral bicarbonate therapy in older CKD patients with acidosis.

### Trial objectives

The primary objective of the BiCARB trial is to determine whether oral bicarbonate therapy improves physical function compared to placebo in older people with CKD and mild acidosis. The secondary objectives are a) to determine whether oral bicarbonate therapy improves health-related quality of life compared to placebo; to compare the impact of oral bicarbonate therapy against placebo on biochemical markers of chronic kidney disease, c) to assess whether use of oral bicarbonate therapy is associated with an excess of adverse events compared with placebo; d) to estimate the cost-effectiveness of using oral bicarbonate therapy compared with placebo, and e) to assess the effect of oral bicarbonate therapy compared with placebo on bone turnover and vascular health as assessed by biochemical markers.

## Methods/Design

### Study design

The trial design is a randomised, double-blind, parallel group, placebo controlled trial, analysed by intention to treat. The treatment and follow-up will last 2 years for each participant. Key outcomes will include measures of physical function, quality of life, progression of renal disease, bone turnover, vascular health and adverse events. A comprehensive health economic analysis is embedded in the trial.

### Study population

The study will recruit 380 community-dwelling participants aged 60 and older with GFR <30 mL/min/1.73 m^2^, not on renal replacement therapy, with a serum bicarbonate concentration of <22 mmol/L (the lower limit of the reference range in most UK centres). Potential participants will be recruited from both primary care and through secondary care sites, for example, renal clinics, Medicine for the Elderly clinics, general Medicine clinics, diabetes clinics, hypertension clinics and cardiovascular clinics at each site. At each centre, the local investigator and renal physicians, renal specialist nurses, geriatricians, cardiologists and diabetologists will be assisted by the study research nurses to identify potential participants. Where local patient registries are kept, these will be exploited to search for potentially suitable participants.

Primary care recruitment will be carried out with the assistance of the primary care research networks in Scotland (SPCRN) and England (CRN). Network involvement via the Ageing and Renal UKCRN specialty groups will assist with recruitment, access to patients and access to network research nurse time. Table 1 gives the inclusion and exclusion criteria for the trial. Potential participants already taking oral bicarbonate therapy will have the option to enter the trial after a 3-month washout period. Written informed consent will be obtained from all participants prior to enrolment. Research ethics committee approval has been given by the East of Scotland Research Ethics Committee (ref 12/ES/0023), the trial is approved by the UK Medicines and Healthcare Regulatory Agency (EudraCT number 2011-005271-16) and the trial is registered at www.isrctn.com (ISRCTN09486651). The trial is funded by the UK NIHR Health Technology Assessment programme (ref: 10/71/01). The Sponsor is Tayside Academic Health Sciences Centre, a joint initiative of the University of Dundee and NHS Tayside, and trial management is via Tayside Clinical Trials Unit (UKCTU number 49).

**Table 1:** Inclusion and exclusion criteria for the BiCARB trial

*Inclusion criteria:*
Participant is willing and able to give informed consent for participation in the study
Male or female aged 60 years or above
Last known estimated glomerular filtration rate (eGFR) <30 ml/min/1.73 m^2^ by MDRD4 equation
Serum bicarbonate <22 mmol/L
Able (in the investigator’s opinion) and willing to comply with all study requirements.
*Exclusion criteria:*
Severe cognitive impairment precluding written informed consent
Already taking bicarbonate therapy unless a 3-month washout period is planned
Documented renal tubular acidosis (likely to require bicarbonate, often in very large doses)
On renal replacement therapy (haemodialysis or peritoneal dialysis)
Anticipated to start renal replacement therapy within 3 months
Participant who is terminally ill, as defined as less than 3-months expected survival
Decompensated chronic heart failure (to ensure that fluid overload is not exacerbated by the additional sodium load from the intervention)
Bisphosphonate therapy (to avoid obscuring bone turnover effects; patients with eGFR <30 mL/min/1.73 m^2^ should not usually be taking bisphosphonates as this is a listed contraindication)
Uncontrolled hypertension at screening visit (BP >150/90 mmHg despite use of four agents), unless evidence from home or 24-hour blood pressure monitoring that blood pressure is usually controlled.
Subject participated in another clinical trial (other than observational studies and registries) concurrently or within 30 days prior to screening for entry into this study
Participant has a known allergy to sodium bicarbonate or lactose

### Intervention

The study medication will be administered as oral tablets. Placebo and active tablets will be identical in appearance. Bicarbonate tablets will contain 500 mg of sodium bicarbonate (6 mmol sodium and 6 mmol bicarbonate); placebo tablets will be composed of lactose. The Good Medical Practice (GMP)-compliant investigational medicinal product manufacturer (Legosan AB, Kumla, Sweden) will prepare and bottle matching active and placebo tablets for the trial. Participants will commence therapy at a dose of 500 mg of oral sodium bicarbonate or placebo three times per day; reflecting a common starting dose in clinical practice. At 3 months, if serum bicarbonate is found to be <22 mmol/L, the dose will be increased to 1 g three times per day or placebo. This is equivalent to six tablets per day; larger doses are likely to worsen adherence to therapy. This dose is higher (3 g versus 1.8 g) than that used in a previous small RCT [13]; it is therefore likely to produce a larger increment in bicarbonate at an earlier time point, thus enhancing the ability of our trial to demonstrate beneficial effects on outcome parameters.

Randomisation will be via a centrally controlled web-based Good Clinical Practice (GCP)-compliant randomisation system, run by Tayside Clinical Trials Unit (TCTU). Investigators at each site will access the web-based system to allocate each participant at the end of the baseline visit. Randomisation will be stratified by site. To ensure balanced assignment across critical variables, a minimisation algorithm will be employed, using baseline age, sex and GFR category to balance allocation across trial arms.

To ensure that medication adherence is maximised, we will employ a combination of written information about the study medication and why taking it is important, together with aide memoirs including BiCARB-branded mugs and fridge magnets. In the second year of follow-up, regular telephone contact (at 15 and 21 months) will be made to remind participants about the importance of medication adherence. Interventions incorporating these components have been shown in a previous Cochrane review to enhance adherence [26]. If the study drug needs to be stopped or the patient wishes to stop they can remain in the study in order to perform an intention-to treat-analysis.

### Outcomes

The primary outcome for the trial is the between-group difference in change for the Short Physical Performance Battery (SPPB) between baseline and 12 months. The SPPB is a validated measure of lower limb function that reflects everyday activity. It is a powerful predictor of subsequent disability, institutionalisation and death in older people [27–29], and has more data validating its use in older people than any other measure of physical function. Secondary outcomes will include SPPB at other time points, along with measures of physical function (grip strength as measure of upper limb function, and six minute walk as a measure of endurance capacity), quality of life (generic, measured using the EQ-5D tool, and disease-specific, measured using the KDQoL tool [30]), anthropometry, renal function and bicarbonate concentrations, markers of bone turnover, calcium and phosphate metabolism, blood pressure and B-type natriuretic peptide. Key outcomes are listed in Table 2.

**Table 2:** Outcome measures and measurement points

OUTCOME	TIMELINE						
	Screening	Baseline	3 months	6 months	12 months	18 months	24 months
*Informed Consent*	X						
*Inclusion/Exclusion Criteria*	X						
*Medical History/Demographics*	X						
*Adverse Events Recorded*		X	X	X	X	X	X
*Physical function measures*							
Short Physical Performance Battery		X	X	X	X		X
Hand Grip Strength		X	X	X	X		X
Six Minute Walk Test		X	X	X	X		X
*Blood measures*							
Sodium	X	X	X	X	X		X
Potassium	X	X	X	X	X		X
Creatinine	X	X	X	X	X		X
Urea	X	X	X	X	X		X
Bicarbonate	X	X	X	X	X		X
Estimated GFR	X	X	X	X	X		X
Calcium		X	X	X	X		X
Phosphate		X	X	X	X		X
Alkaline phosphatase		X	X	X	X		X
Magnesium		X	X	X	X		X
Albumin		X	X	X	X		X
Haemoglobin		X	X	X	X		X
Thyroid function		X	X	X	X		X
HbA1c		X	X	X	X		X
Total Cholesterol		X	X	X	X		X
Cystatin C		X	X	X	X		X
Study Biobank Samples		X	X	X	X		X
*Urine Sample*							
Protein/creatinine ratio		X	X	X	X		X
Albumin/creatinine ratio		X	X	X	X		X
*Questionnaires*							
EQ-5D		X	X	X	X		X
KDQoL		X	X	X	X		X
Health Diary		X	X	X	X		X
Falls Diary		X^a^	X	X	X	X	X
*Anthropometric Measurements*							
Mid-arm Circumference		X	X	X	X		X
Triceps Skin Fold Thickness		X	X	X	X		X
Mid-thigh Circumference		X	X	X	X		X
Height		X					
Weight		X	X	X	X		X
*Vascular measures*							
Blood pressure and pulse	X	X	X	X	X		X
B-type natriuretic peptide		X			X		X
*Bone and calcium metabolism*							
Calcium		X	X	X	X		X
Phosphate		X	X	X	X		X
Parathyroid hormone		X			X		X
25-hydroxyvitamin D		X			X		X
1,25 hydroxyvitamin D		X			X		X
Tartrate-resistant acid phosphatase-5b		X			X		X
Bone-specific alkaline phosphatase		X			X		X

^a^administered at baseline but not recorded until 3 months

Adverse events and medication changes will be sought at each study visit, as will data on hospitalisation, death (all-cause, cardiovascular and renal), commencement of renal replacement therapy, fractures, primary care and secondary care outpatient visits via participant health-utilisation diaries. Adherence to medication will be assessed by tablet count on bottles returned to the trial pharmacy. Detailed information on medical history, concomitant medications (including renal and cardiovascular medications such as renin-angiotensin system blockers), social and functional history will be collected at baseline. Participant flow through the trial is outlined in Fig. 1.

### Statistical analysis

Statistical analysis will be performed by intention-to-treat and reported in accordance with the CONSORT statement (www.equator-network.org). The primary analysis will be a between-group comparison of 12 month outcomes, adjusted for baseline values of the outcome under test via regression analysis. Further adjustment for age, sex and GFR category will also be performed. Secondary analyses will comprise multivariate repeated measures analysis using all available time points, adjusted for baseline differences. A per-protocol secondary analysis will be performed to compare adverse events, and time-to-event analyses for death or onset of renal replacement therapy will be performed using Cox proportional hazards regression models. A two-sided *P* value of 0.05 will be taken as significant for each outcome. The primary outcome will be analysed at 12 months rather than 24 months in order to strike a balance between excessive dropout (expected in this group of older, multi-morbid participants) and sufficient time for the intervention to show an effect.

Pre-specified subgroup analyses will include the following: participants with a SPPB score of <10 (denoting a frailer subgroup) versus those with SPPB ≥10; participants with a baseline serum bicarbonate concentration of >18 mmol/L versus those with serum bicarbonate ≤18 mmol/L; GFR category (4 versus 5); sex, and age >75 years versus age ≤75 years. Missing data for the primary analysis will be handled using multiple imputation, provided that the assumption of missing at random is met. Missing data in the repeated measures analyses will be handled using generalised estimating equation approaches to maximise the use of participants with incomplete data.

### Data analysis of health economic data

The economic analysis will estimate the healthcare costs and quality of life associated with provision of bicarbonate therapy relative to usual care (that is, usual healthcare management without the addition of bicarbonate therapy) over the time period of the trial (12 months for the main analysis). It will determine the magnitude of the difference in healthcare costs and quality of life between the two treatments. Healthcare costs will include the type and duration of hospital admissions, frequency of visits to hospital for outpatient attendances, and other visits to/from relevant healthcare professionals (for example, general practitioners, nurse practitioners, and physiotherapists) at baseline and over 12 months. Quality of life will be measured with the EQ-5D instrument over an equivalent time period, and responses converted to a single summary score using published UK quality of life weights, to produce a Quality Adjusted Life Year, (‘QALY’) value. In addition to QALYs, secondary analyses will include the measurement of well-being and life satisfaction. Cost effectiveness acceptability curves will be employed to show the probability that bicarbonate therapy is cost effective for different values of willingness to pay per additional QALY [31]. These will be developed using the quality of life gain and life expectancy change estimates from the ANCOVA and repeated measures analysis. Cost differences will be modelled using generalized linear modelling, with appropriate adjustment for skew and other baseline differences between groups. In addition, non-parametric bootstrap methods will be used for calculating confidence intervals around cost and QALY differences. Sensitivity analysis will be undertaken for uncertain parameters, such as alternative centre specific costs per bed day and alternative age-adjusted quality of life weights.

A detailed statistical analysis plan will be agreed on before the end of data entry and before the treatment code is broken.

### Sample size calculation

We plan to randomize a total of 380 participants: 190 to bicarbonate and 190 to placebo. The clinically important difference (CID) for the SPPB is a 1-point difference. Assuming a SD of 2.6 as found in previous work [32], we would require 143 patients per group to give 90 % power to detect this difference at a two-sided alpha of 0.05. For the EQ-5D, the minimum CID is 0.074 [33]. To detect this with two-sided alpha = 0.05 and power of 90 %, assuming a SD of change of 0.2, as found in our previous studies, our study requires 154 patients per group. We anticipate a dropout rate of 10 % per 6 months, due to the high rates of expected death and illness in this multi-morbid, older group of participants; the calculated sample size accounts for this dropout.

Progression to dialysis in a previous trial of bicarbonate therapy [13] was lower in patients allocated to bicarbonate (7 %) than in the control arm (33 %). The proposed sample size will have 90 % power to successfully detect a more conservative difference of 7 % in the bicarbonate arm and 18 % in the control arm at 2 years. Assuming a 10 % loss to follow-up every 6 months (a pessimistic estimate, based on previous medication trials in frail older people in our centre), we would require 380 patients to ensure adequate power for the primary outcome and the EQ-5D at 12 months. This rate of attrition would leave 250 evaluable patients at 2 years, which will give additional power to detect smaller changes using repeated measures analyses.

## Discussion

In many areas of medicine, older people are underrepresented in clinical trials [34], and even when included, older people in trials often lack the frailty and multi-morbidity that characterise typical patients treated in clinical practice. Hence the evidence base for many interventions is not generalizable to typical older people.

Although oral bicarbonate is widely used to correct acidosis in advanced CKD, this is not underpinned by trial evidence, and real uncertainty exists regarding the balance of benefit and risk for this intervention. As most patients with CKD are old, and many are frail, it is critical that trials testing such interventions enrol typical patients and use outcome measures that are relevant to older people. Few older people with even advanced CKD will progress to end-stage renal disease; the risk of death from cardiovascular disease or infection often supervenes long before the need for renal replacement therapy. The range of outcomes selected for this trial will allow an estimation of overall net benefit or harm across a range of disease outcomes including renal, bone and vascular disease, as well as focusing on outcomes that are important to patients (function and quality of life) and policymakers (cost-utility analysis).

Three other randomised controlled trials with a projected sample size >100 are either planned or in progress in this area. One, a multicentre trial based in Italy [35], aims to randomise 600 patients aged <80 years with GFR categories 3b or 4 and serum bicarbonate ≥18 mmol/L, and will compare usual care with a strategy of maintenance of serum bicarbonate >24 mmol/L but <28 mmol/L for 3 years, using doses of up to 4 g (48 mmol) of sodium bicarbonate per day. The trial is randomised but is not blinded. The primary outcome is doubling of serum creatinine, with all-cause mortality and requirement for renal replacement therapy as secondary outcomes. This trial will be well equipped to examine renal outcomes but will not focus on physical function or quality of life, which are key outcomes for older patients.

The second trial underway (NCT01452412; https://clinicaltrials.gov), based in the United States, aims to recruit 150 patients with GFR categories 3b or 4 and serum bicarbonate 20 to 25 mmol/L, who will receive 0.4 mmol/Kg/day of oral bicarbonate or placebo for 1 year. A range of outcomes including insulin resistance, grip strength, lower limb function, wrist bone mineral density, renal function and proteinuria will be measured. This trial should give useful information across a range of disease states; the different population (milder acidosis and less severe CKD) should complement the results from our trial, but will not focus on health economic outcomes.

The third trial [36] is a single centre study being conducted in Austria. It aims to recruit 200 patients with GFR categories 3 or 4 and serum bicarbonate <21 mmol/L. Participants will be randomised to one of two treatment strategies - either high-dose oral bicarbonate supplementation (1.7 g to 5 g (60 mmol) per day) titrated to keep serum bicarbonate >24 mmol/L, or a strategy of withholding bicarbonate unless serum bicarbonate falls below 19 mmol/L, when low-dose oral bicarbonate will be administered with a target serum bicarbonate of 20 mmol/L. The primary outcome will be the effect of therapy on estimated GFR; secondary outcomes will include markers of bone metabolism, the need for renal replacement therapy, and death. Treatment and follow up will be for 2 years. The population under study in this trial will have less severe CKD than the BiCARB trial population, outcomes are predominantly focused on renal function, and the trial compares two strategies of bicarbonate replacement, rather than bicarbonate versus placebo as in the BiCARB trial.

In conclusion, the trial we describe should provide much-needed evidence on the net risk-benefit balance for this commonly used therapy in typical older patients with advanced CKD. As such, the results should be directly relevant to the care of this patient group and hence be of value to clinicians and their patients, guideline developers and policymakers.

## Trial status

At the time of writing, approximately 220 of the projected 380 participants have been randomised, and results are expected in late 2018.

## Abbreviations

ANCOVA: analysis of covariance
CID: clinically important difference
CKD: chronic kidney disease
eGFR: estimated glomerular filtration rate
EQ-5D: EuroQoL 5D
GCP: good clinical practice
GMP: good manufacturing practice
KDIGO: Kidney Disease: Improving Global Outcomes
KDQoL: Kidney Disease Quality of Life
NICE: National Institute for Health and Care Excellence
NIHR: National Institute of Health Research
QALY: quality adjusted life years
RCT: randomised controlled trial
SIGN: Scottish Intercollegiate Guidelines Network
SPCRN: Scottish Primary Care Research Network
SPPB: Short Physical Performance Battery
TCTU: Tayside Clinical Trials Unit
UKCRN: United Kingdom Clinical Research Network
UKCTU: United Kingdom Clinical Trials Unit
